# Supplementary material for: Validity of Effective Potentials in Crowded Solutions of Linear and Ring Polymers with Reversible Bonds
Source: Macromolecules. 2022 Mar 24;55(7):2659–74. doi: 10.1021/acs.macromol.1c02610 (PMC9011144; doi:10.1021/acs.macromol.1c02610)
Supplement: Supplementary file 1 — ma1c02610_si_001.pdf [file ma1c02610_si_001.pdf]

Supporting Information: On the Validity of Effective Potentials in Crowded Solutions of Linear and Ring Polymers with Reversible Bonds

Mariarita Paciolla,† Christos N. Likos,‡ and Angel J. Moreno*,†,¶

Centro de Física de Materiales (CSIC, UPV/EHU) and Materials Physics Center MPC, Paseo Manuel de Lardizabal 5, 20018 San Sebastián, Spain, Faculty of Physics, University of Vienna, Boltzmanngasse 5, A-1090 Vienna, Austria, and Donostia International Physics Center, Paseo Manuel de Lardizabal 4, 20018 San Sebastian, Spain

E-mail: angeljose.moreno@ehu.es

*To whom correspondence should be addressed

†Centro de Física de Materiales (CSIC, UPV/EHU) and Materials Physics Center MPC, Paseo Manuel de Lardizabal 5, 20018 San Sebastián, Spain

‡Faculty of Physics, University of Vienna, Boltzmanngasse 5, A-1090 Vienna, Austria

¶Donostia International Physics Center, Paseo Manuel de Lardizabal 4, 20018 San Sebastian, Spain

Derivation of TPR equations

It can be shown[1] that the grand potential of an inhomogeneous fluid in equilibrium with a bulk reservoir that fixes the chemical potential μ is a unique functional of the equilibrium single-particle density $\rho(\mathbf{r})$. DFT enters in the computation of $\rho(\mathbf{r})$, which can be obtained by minimising the grand potential functional:[2]

$$\Omega[\rho] = F_{\text{ideal}}[\rho] + F_{\text{ex}}[\rho] + \int \rho(\mathbf{r}) V_{\text{eff}}(\mathbf{r}) d^3r - \mu \int \rho(\mathbf{r}) d^3r, \tag{1}$$

The first sum on the right side is the Helmholtz free energy $F[\rho]$ with a well-known ideal contribution equal to:

$$F_{\text{ideal}}[\rho] = \beta^{-1} \int \rho(\mathbf{r})[\ln(\rho(\mathbf{r})\Lambda^3) - 1] d^3r. \tag{2}$$

In this expression $\beta = 1/k_{\text{B}}T$ and $\Lambda = \sqrt{2\pi\beta\hbar^2/m}$ is the thermal de Broglie wavelength. In order to compute the excess contribution to the free energy functional we use the mean field approximation:[3,4]

$$F_{\text{ex}}[\rho] = \frac{1}{2} \iint \rho(\mathbf{r})\rho(\mathbf{r}') V_{\text{eff}}(|\mathbf{r} - \mathbf{r}'|) d^3r \, d^3r'. \tag{3}$$

By substituting Eq. (2) and Eq. (3) in Eq. (1), and by performing the functional derivative, we find:

$$\frac{\delta(\beta\Omega[\rho])}{\delta\rho(\mathbf{r})} = \int \rho(\mathbf{r}')\beta V_{\text{eff}}(|\mathbf{r} - \mathbf{r}'|) d^3r' + \ln(\rho(\mathbf{r})\Lambda^3) + \beta V_{\text{eff}}(|\mathbf{r}|) - \beta\mu, \tag{4}$$

The expression for the chemical potential is obtained by computing the Helmholtz free energy in the bulk density ρ_{b}:

$$\mu = \frac{\partial(\beta F[\rho_{\text{b}}])}{\partial N} = \ln(\rho_{\text{b}}\Lambda^3) + \rho_{\text{b}} \int \beta V_{\text{eff}}(|\mathbf{r}|) d^3r, \tag{5}$$

By substituting Eq. (5) in Eq. (4) and noting that the integral involves a convolution of V_{eff} and ρ,

we obtain

$$\ln(\rho(|\mathbf{r}|)/\rho_{\mathrm{b}}) = -\beta(V_{\mathrm{eff}} * \rho)(|\mathbf{r}|) - \beta V_{\mathrm{eff}}(|\mathbf{r}|) + \rho_{\mathrm{b}} \int \beta V_{\mathrm{eff}}(|\mathbf{r}|) d^3 r, \tag{6}$$

where the symbol $*$ denotes the convolution. By introducing a new variable[2] $\Delta\rho(\mathbf{r}) = \rho(\mathbf{r}) - \rho_{\mathrm{b}}$, which decays to 0 at long distances, and by substituting it in Eq. (6), the final expression for the density profile is obtained:

$$\Delta\rho(|\mathbf{r}|) = \rho_{\mathrm{b}}(\exp[-\beta(V_{\mathrm{eff}} * \Delta\rho)(|\mathbf{r}|) - \beta V_{\mathrm{eff}}(|\mathbf{r}|)] - 1), \tag{7}$$

and as mentioned in the article the radial distribution function is just calculated as $g(|\mathbf{r}|) = \rho(|\mathbf{r}|)/\rho_{\mathrm{b}}$.

In the case of a binary mixture, there are 2 species with densities $\rho_i(\mathbf{r})$ that interact through $V_{ij}(|\mathbf{r}|)$, with $i, j = 1, 2$. The corresponding Helmholtz free energy for this system is given by:

$$F[\rho_1, \rho_2] = \sum_{i=1}^{2} \int \rho_i(\mathbf{r})[\ln(\Lambda_i^3 \rho_i(\mathbf{r})) - 1] d^3 r + \frac{1}{2} \sum_{i=1}^{2} \sum_{j=1}^{2} \int d^3 r \int d^3 r' \rho_i(\mathbf{r}) \rho_j(\mathbf{r}') V_{ij}(|\mathbf{r} - \mathbf{r}'|). \tag{8}$$

Thus, if a particle of the species 1 is fixed in the origin, the grand potential functional reads as:

$$\Omega[\rho_1, \rho_2] = F[\rho_1, \rho_2] + \int \rho_1(\mathbf{r}) V_{11}(|\mathbf{r}|) d^3 r + \int \rho_2(\mathbf{r}) V_{12}(|\mathbf{r}|) d^3 r - \mu_1 \int \rho_1(\mathbf{r}) d^3 r - \mu_2 \int \rho_2(\mathbf{r}) d^3 r. \tag{9}$$

Then by using the same procedure as before it is possible to obtain 2 equations that need to be solved iteratively for determining $g_{11}(|\mathbf{r}|)$ and $g_{12}(|\mathbf{r}|)$. Equivalently, by fixing in the origin a particle of species 2 one obtains the expressions for $g_{22}(|\mathbf{r}|)$ and $g_{21}(|\mathbf{r}|)$.

	Linear
all monomers (AM)	$L_{\text{box}} = 175, \quad N_{\text{lin}} = 108, \quad \rho = 0.004, \quad \rho/\rho^* = 0.16$
	$L_{\text{box}} = 77, \quad N_{\text{lin}} = 108, \quad \rho = 0.047, \quad \rho/\rho^* = 1.85$
	$L_{\text{box}} = 64, \quad N_{\text{lin}} = 108, \quad \rho = 0.082, \quad \rho/\rho^* = 3.23$
	$L_{\text{box}} = 55, \quad N_{\text{lin}} = 108, \quad \rho = 0.129, \quad \rho/\rho^* = 5.08$
	$L_{\text{box}} = 50, \quad N_{\text{lin}} = 108, \quad \rho = 0.173, \quad \rho/\rho^* = 6.77$
effective fluid (EF)	$L_{\text{box}} = 367.5, \quad N_{\text{lin}} = 1000, \quad \rho/\rho^* = 0.16$
	$L_{\text{box}} = 161.7, \quad N_{\text{lin}} = 1000, \quad \rho/\rho^* = 1.85$
	$L_{\text{box}} = 134.4, \quad N_{\text{lin}} = 1000, \quad \rho/\rho^* = 3.23$
	$L_{\text{box}} = 115.5, \quad N_{\text{lin}} = 1000, \quad \rho/\rho^* = 5.08$
	$L_{\text{box}} = 105.0, \quad N_{\text{lin}} = 1000, \quad \rho/\rho^* = 6.77$
	Ring
all monomers (AM)	$L_{\text{box}} = 175, \quad N_{\text{lin}} = 108, \quad \rho = 0.004, \quad \rho/\rho^* = 0.063$
	$L_{\text{box}} = 77, \quad N_{\text{ring}} = 108, \quad \rho = 0.047, \quad \rho/\rho^* = 0.74$
	$L_{\text{box}} = 62, \quad N_{\text{ring}} = 108, \quad \rho = 0.090, \quad \rho/\rho^* = 1.42$
	$L_{\text{box}} = 50, \quad N_{\text{ring}} = 108, \quad \rho = 0.173, \quad \rho/\rho^* = 2.71$
	$L_{\text{box}} = 43, \quad N_{\text{ring}} = 108, \quad \rho = 0.272, \quad \rho/\rho^* = 4.26$
effective fluid (EF)	$L_{\text{box}} = 367.5, \quad N_{\text{ring}} = 1000, \quad \rho/\rho^* = 0.063$
	$L_{\text{box}} = 161.7, \quad N_{\text{ring}} = 1000, \quad \rho/\rho^* = 0.74$
	$L_{\text{box}} = 130.2, \quad N_{\text{ring}} = 1000, \quad \rho/\rho^* = 1.42$
	$L_{\text{box}} = 105.0, \quad N_{\text{ring}} = 1000, \quad \rho/\rho^* = 2.71$
	$L_{\text{box}} = 90.3, \quad N_{\text{ring}} = 1000, \quad \rho/\rho^* = 4.26$

Table S1. Simulation parameters of the solutions (all monomers and effective fluid) of linear and ring polymers with reversible bonds: box size (L_{box}), number of polymers ($N_{\text{lin}}, N_{\text{ring}}$), absolute ($\rho$) and reduced ($\rho/\rho^*$) concentration of monomers.

	Linear-Linear
all monomers (AM)	$L_{\mathrm{box}} = 175,\quad N_{\mathrm{lin},1} = 54,\quad N_{\mathrm{lin},2} = 54,\quad \rho = 0.004,\quad \rho/\rho^* = 0.16$<br>$L_{\mathrm{box}} = 77,\quad N_{\mathrm{lin},1} = 54,\quad N_{\mathrm{lin},2} = 54,\quad \rho = 0.047,\quad \rho/\rho^* = 1.85$<br>$L_{\mathrm{box}} = 64,\quad N_{\mathrm{lin},1} = 54,\quad N_{\mathrm{lin},2} = 54,\quad \rho = 0.082,\quad \rho/\rho^* = 3.23$<br>$L_{\mathrm{box}} = 55,\quad N_{\mathrm{lin},1} = 54,\quad N_{\mathrm{lin},2} = 54,\quad \rho = 0.129,\quad \rho/\rho^* = 5.08$<br>$L_{\mathrm{box}} = 50,\quad N_{\mathrm{lin},1} = 54,\quad N_{\mathrm{lin},2} = 54,\quad \rho = 0.173,\quad \rho/\rho^* = 6.77$
effective fluid (EF)	$L_{\mathrm{box}} = 367.5,\quad N_{\mathrm{lin},1} = 500,\quad N_{\mathrm{lin},2} = 500,\quad \rho/\rho^* = 0.16$<br>$L_{\mathrm{box}} = 161.7,\quad N_{\mathrm{lin},1} = 500,\quad N_{\mathrm{lin},2} = 500,\quad \rho/\rho^* = 1.85$<br>$L_{\mathrm{box}} = 134.4,\quad N_{\mathrm{lin},1} = 500,\quad N_{\mathrm{lin},2} = 500,\quad \rho/\rho^* = 3.23$<br>$L_{\mathrm{box}} = 115.5,\quad N_{\mathrm{lin},1} = 500,\quad N_{\mathrm{lin},2} = 500,\quad \rho/\rho^* = 5.08$<br>$L_{\mathrm{box}} = 105.0,\quad N_{\mathrm{lin},1} = 500,\quad N_{\mathrm{lin},2} = 500,\quad \rho/\rho^* = 6.77$
	Linear-Ring
all monomers (AM)	$L_{\mathrm{box}} = 175,\quad N_{\mathrm{lin}} = 54,\quad N_{\mathrm{ring}} = 54,\quad \rho = 0.004,\quad \rho/\rho^* = 0.11$<br>$L_{\mathrm{box}} = 77,\quad N_{\mathrm{lin}} = 54,\quad N_{\mathrm{ring}} = 54,\quad \rho = 0.047,\quad \rho/\rho^* = 1.30$<br>$L_{\mathrm{box}} = 62,\quad N_{\mathrm{lin}} = 54,\quad N_{\mathrm{ring}} = 54,\quad \rho = 0.090,\quad \rho/\rho^* = 2.49$<br>$L_{\mathrm{box}} = 50,\quad N_{\mathrm{lin}} = 54,\quad N_{\mathrm{ring}} = 54,\quad \rho = 0.173,\quad \rho/\rho^* = 4.74$<br>$L_{\mathrm{box}} = 43,\quad N_{\mathrm{lin}} = 54,\quad N_{\mathrm{ring}} = 54,\quad \rho = 0.272,\quad \rho/\rho^* = 7.45$
efective fluid (EF)	$L_{\mathrm{box}} = 367.5,\quad N_{\mathrm{lin}} = 500,\quad N_{\mathrm{ring}} = 500,\quad \rho/\rho^* = 0.11$<br>$L_{\mathrm{box}} = 161.7,\quad N_{\mathrm{lin}} = 500,\quad N_{\mathrm{ring}} = 500,\quad \rho/\rho^* = 1.30$<br>$L_{\mathrm{box}} = 130.2,\quad N_{\mathrm{lin}} = 500,\quad N_{\mathrm{ring}} = 500,\quad \rho/\rho^* = 2.49$<br>$L_{\mathrm{box}} = 105.0,\quad N_{\mathrm{lin}} = 500,\quad N_{\mathrm{ring}} = 500,\quad \rho/\rho^* = 4.74$<br>$L_{\mathrm{box}} = 90.3,\quad N_{\mathrm{lin}} = 500,\quad N_{\mathrm{ring}} = 500,\quad \rho/\rho^* = 7.45$

Table S2. As Table S1 for the mixtures.

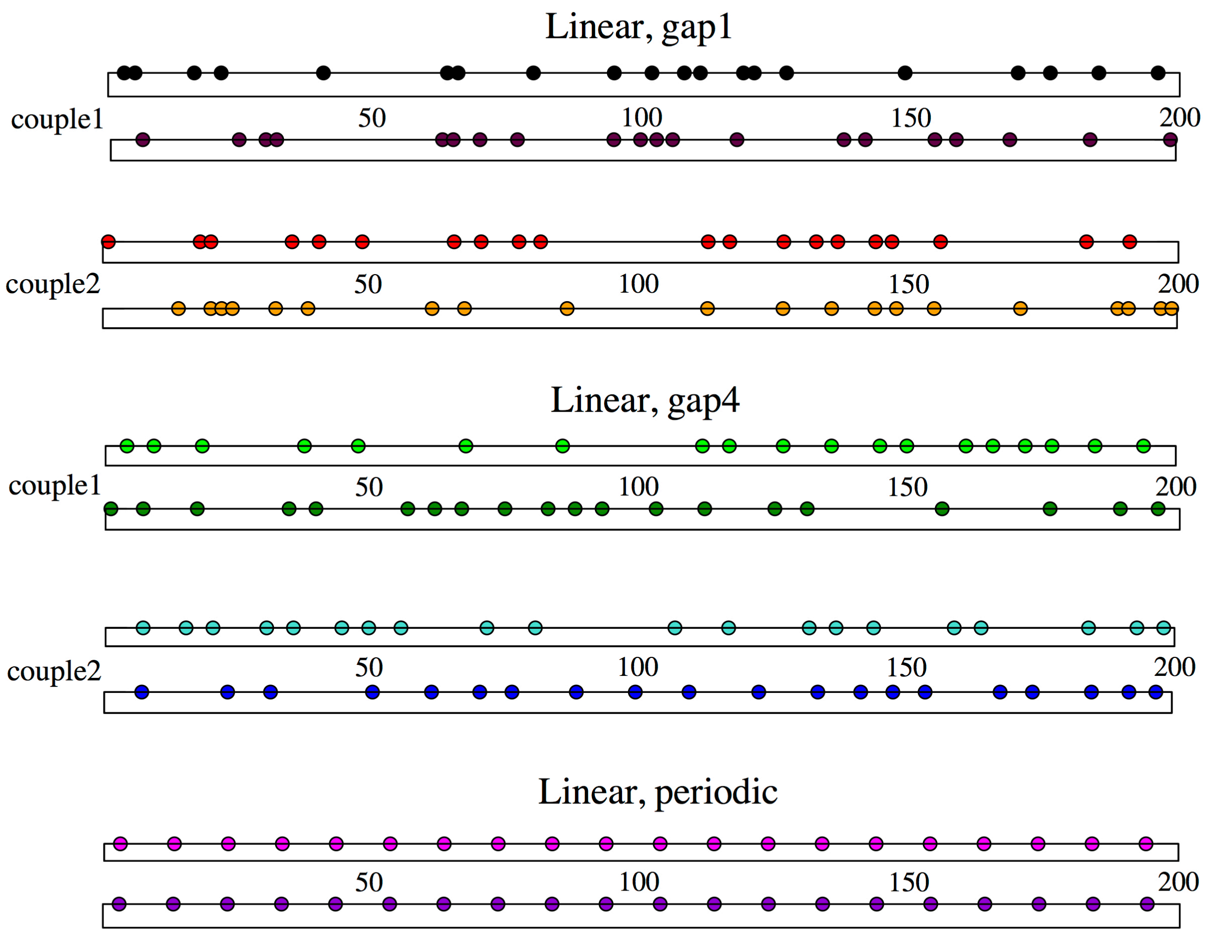

Figure S1. Sequences of the reactive sites along the backbone for the linear chains with reversible bonds used to derive the effective potentials. Each row represents the two backbones of a pair of chains. The chain backbone consists of $N = 200$ beads, 20 of them being reactive and able to form reversible bonds. Only the reactive beads are represented. The sequences are randomly generated with a minimum number of beads between consecutive reactive sites (1 and 4 for gap1 and gap4 cases, respectively). Two couples (1 and 2) are generated for both the gap1 and gap4 cases. A couple of chains with a periodic sequence of reactive sites is also studied (bottom row).

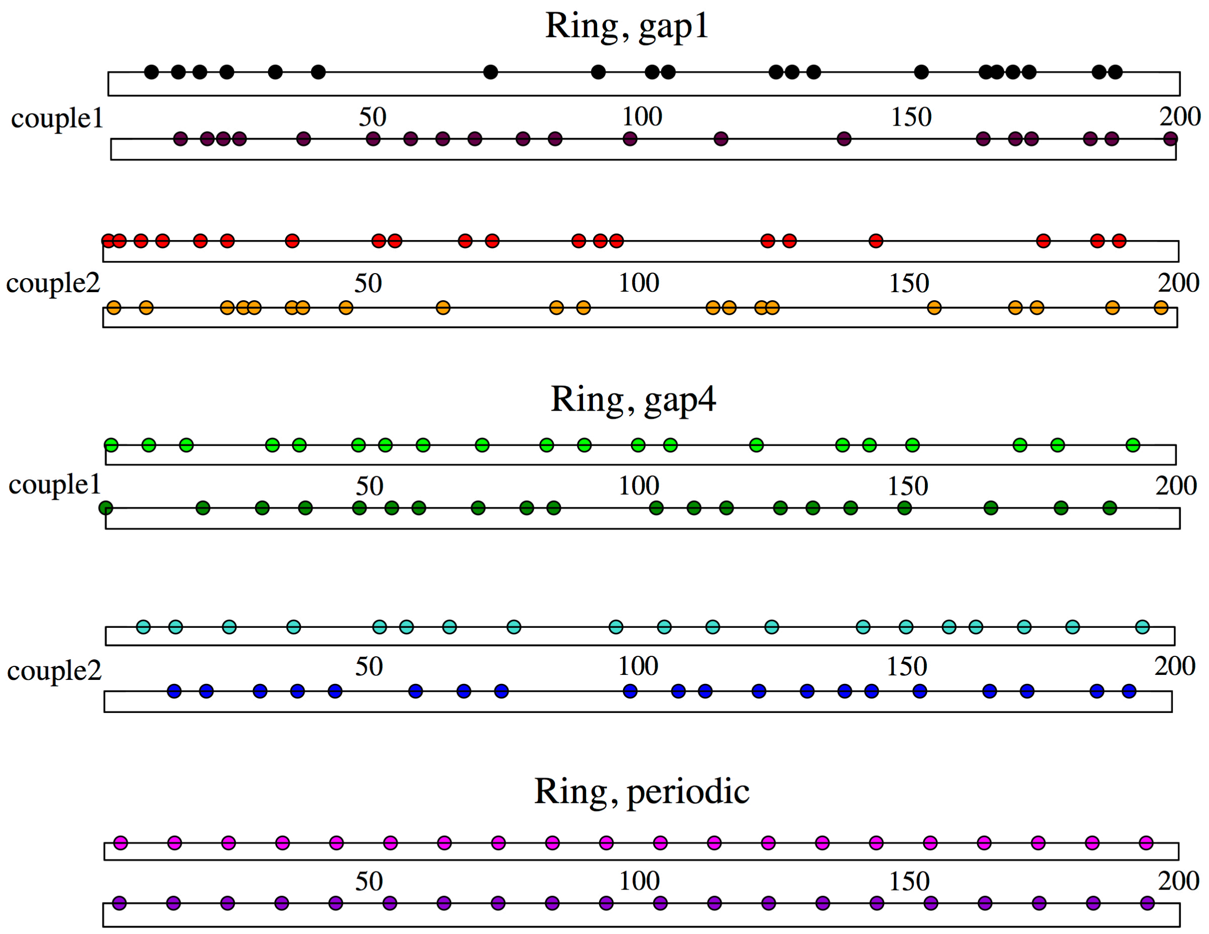

Figure S2. As Figure S1 for the rings with reversible bonds. Although a linear representation of the backbone is used, it should be noted that both ends are mutually and permanently connected because of the ring geometry.

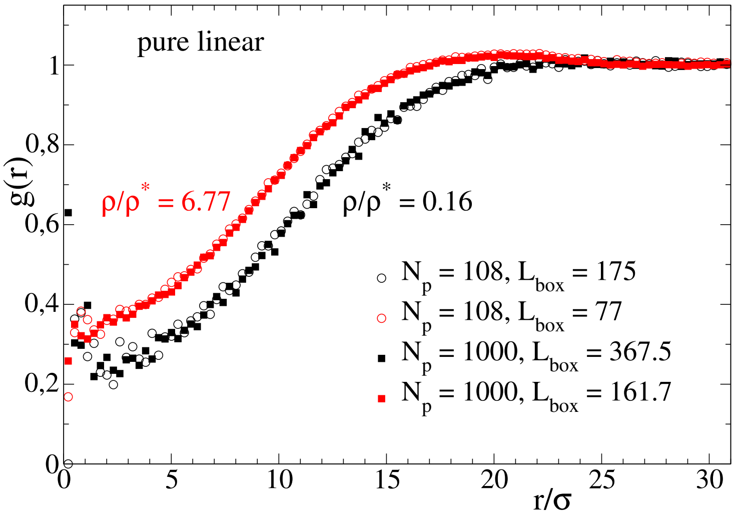

Figure S3. Radial distribution functions of the effective fluid for the system of linear chains with reversible bonds. Data are shown at the lowest and highest investigated concentrations, and in both cases for $N_{\mathrm{p}} = 108$ and 1000 effective particles (with the respective rescaling of the box size to produce the same concentration, see legend). No significant size effects are observed.

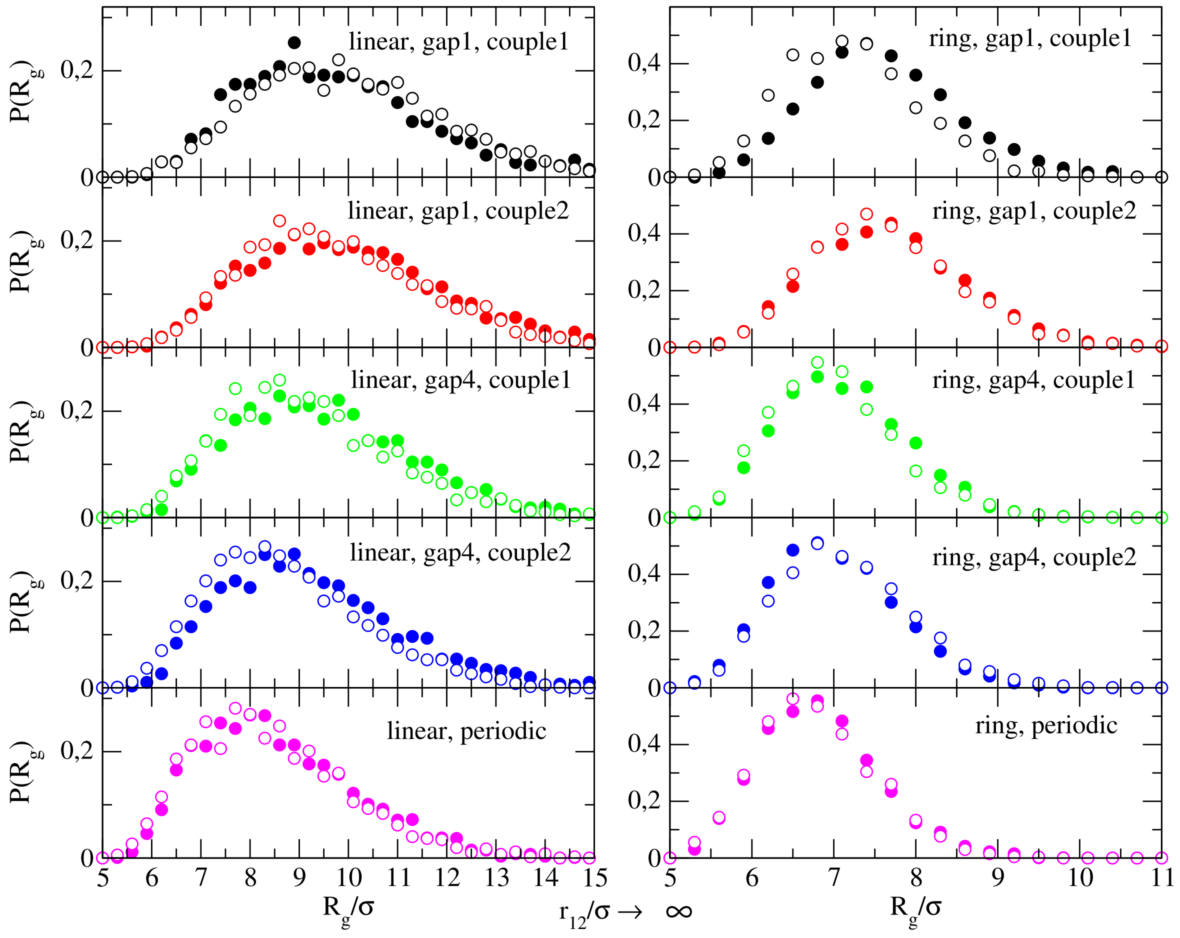

Figure S4. Distribution of the radius of gyration for pairs of polymers at a very large distance (25σ) where there are no mutual contacts and the effective potential is zero. The two sets at each panel are the individual distributions of the two polymers.

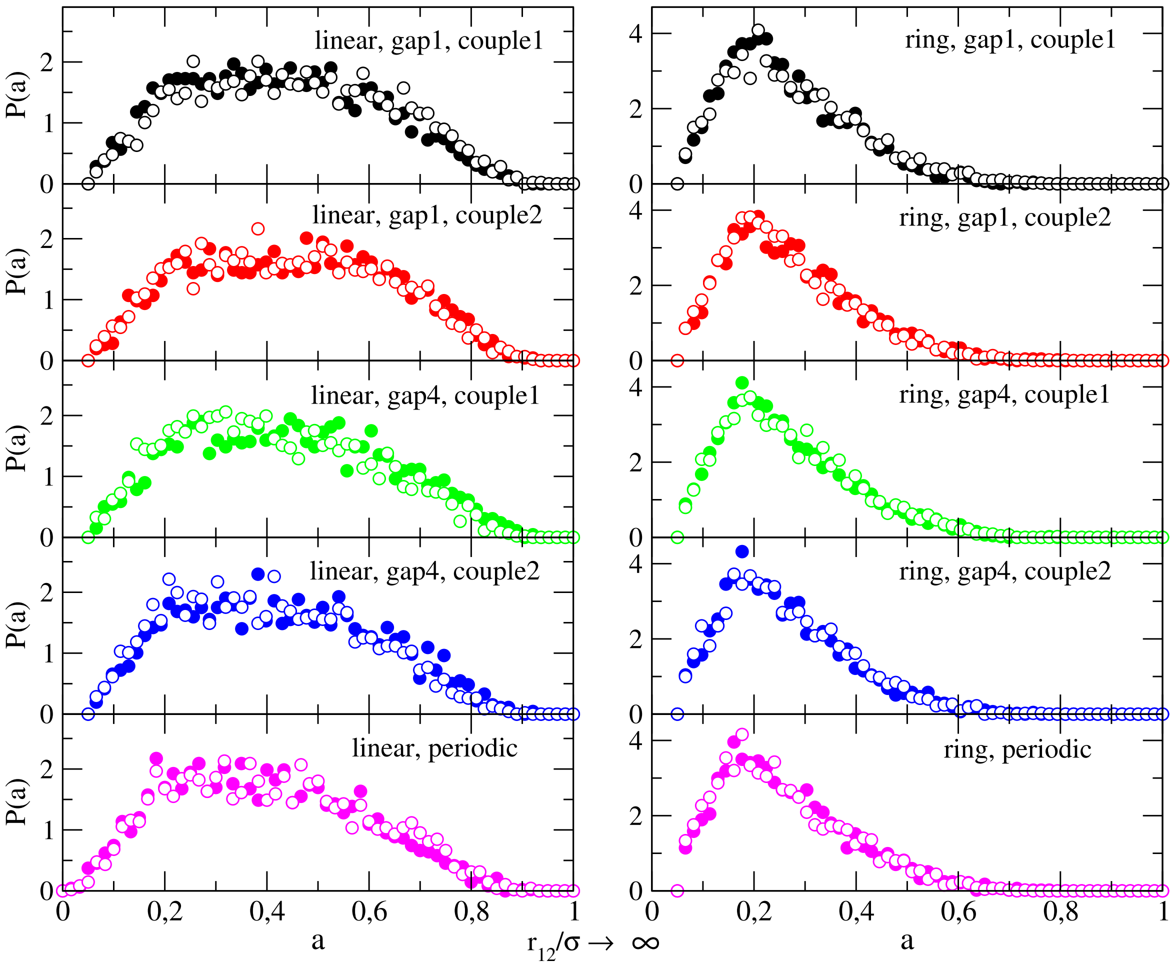

Figure S5. Distribution of the asphericities for pairs of polymers at a very large distance (25σ) where there are no mutual contacts and the effective potential is zero. The two sets at each panel are the individual distributions of the two polymers.

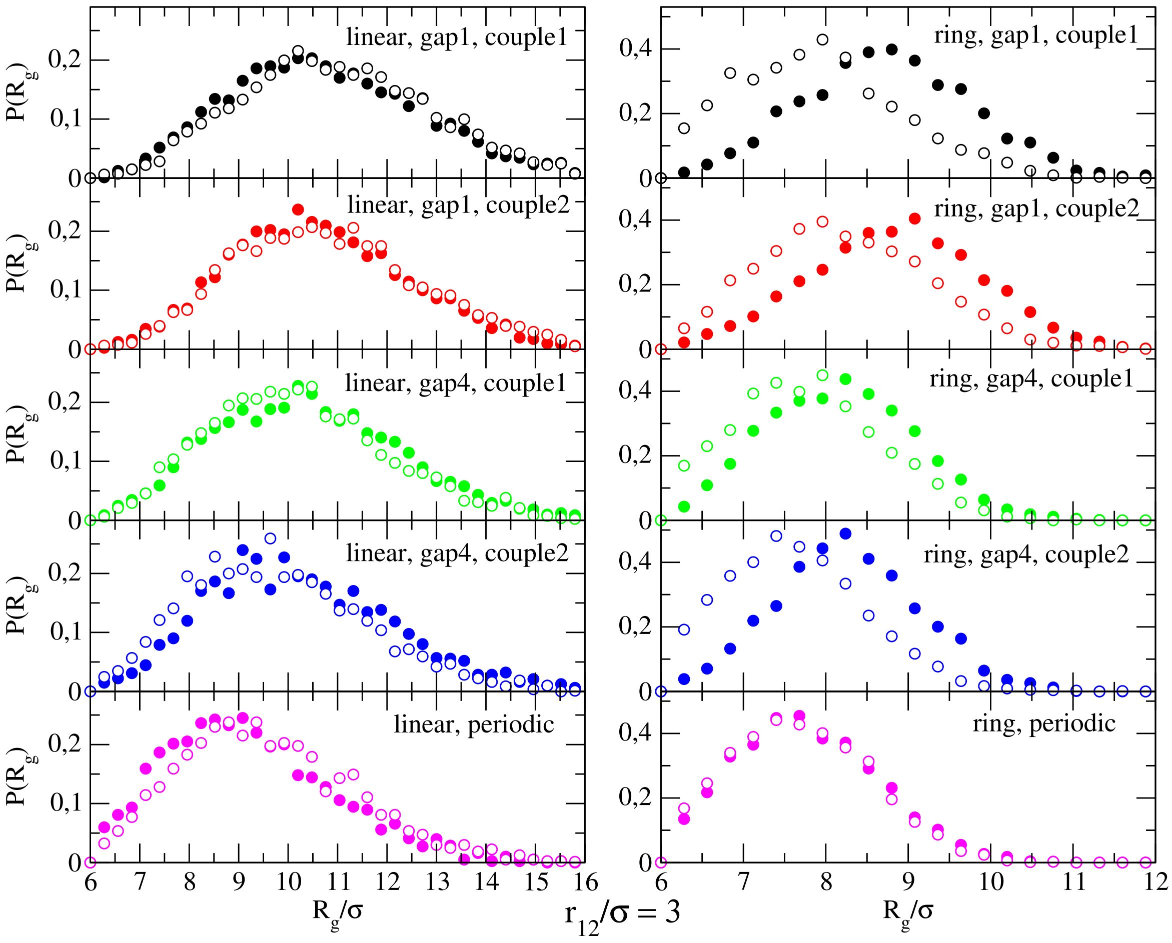

Figure S6. Distribution of the radius of gyration for pairs of polymers at a distance 3σ where the force $\mathbf{F}_{12}$ acting between them has its maximum. The two sets at each panel are the individual distributions of the two polymers.

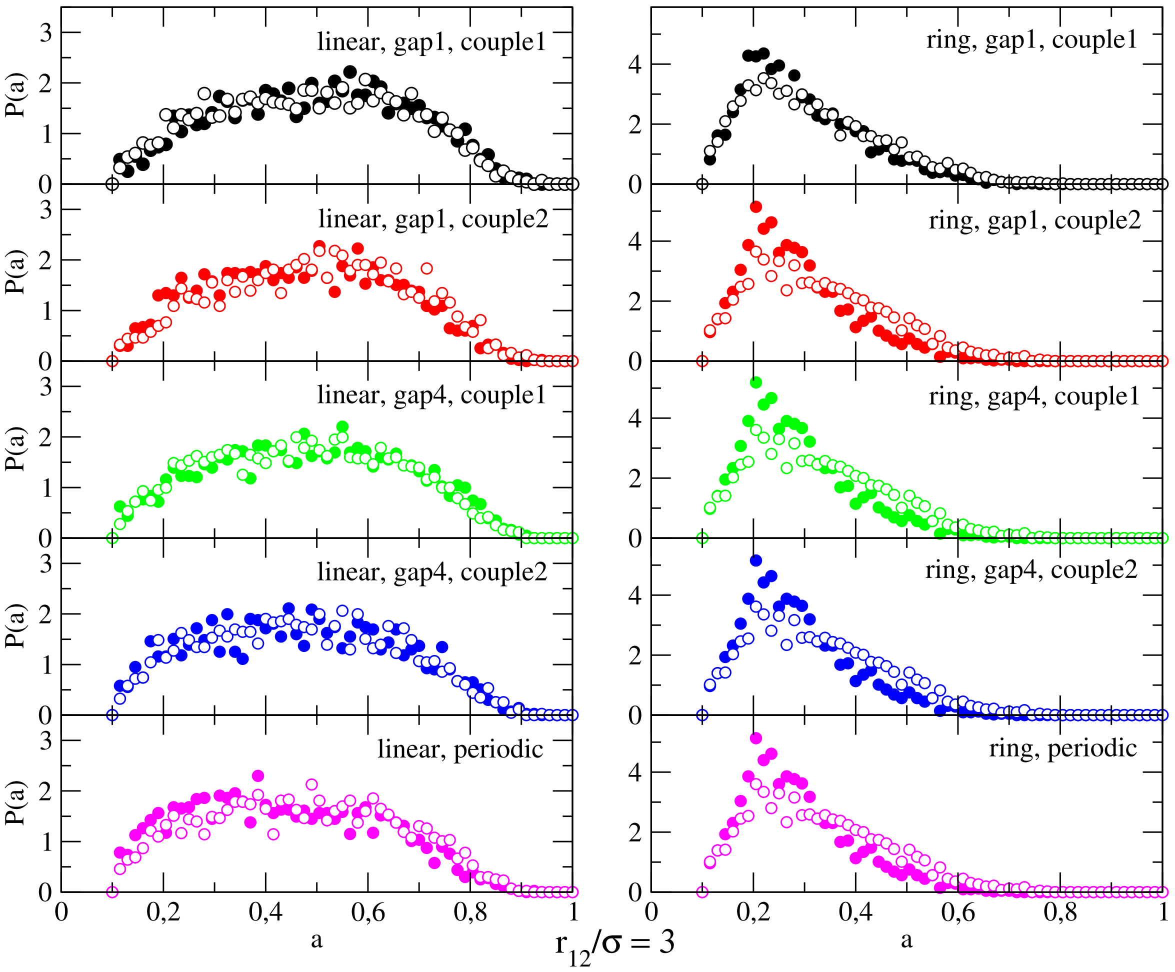

Figure S7. Distribution of the asphericity for pairs of polymers at a distance 3σ where the force $\mathbf{F}_{12}$ acting between them has its maximum. The two sets at each panel are the individual distributions of the two polymers.

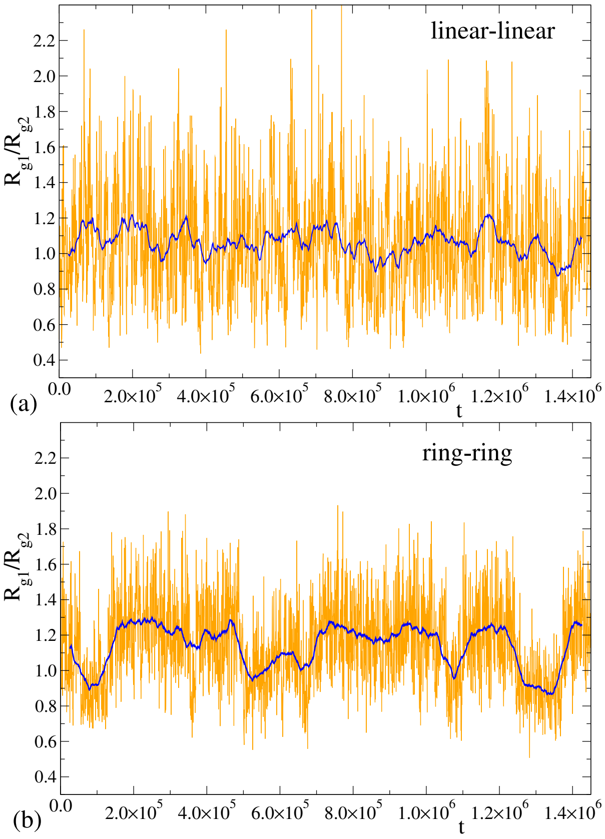

Figure S8. Ratio of the radii of gyration of the two polymers with reversible bonds at a distance 3σ where the force $\mathbf{F}_{12}$ acting between them has its maximum. Panels (a) and (b) correspond to the linear-linear and ring-ring cases, respectively (both cases correspond to the 'gap1, couple1' sequences). Orange curves are the bare data. Blue lines are smoothed data obtained by 100 point-averaging.

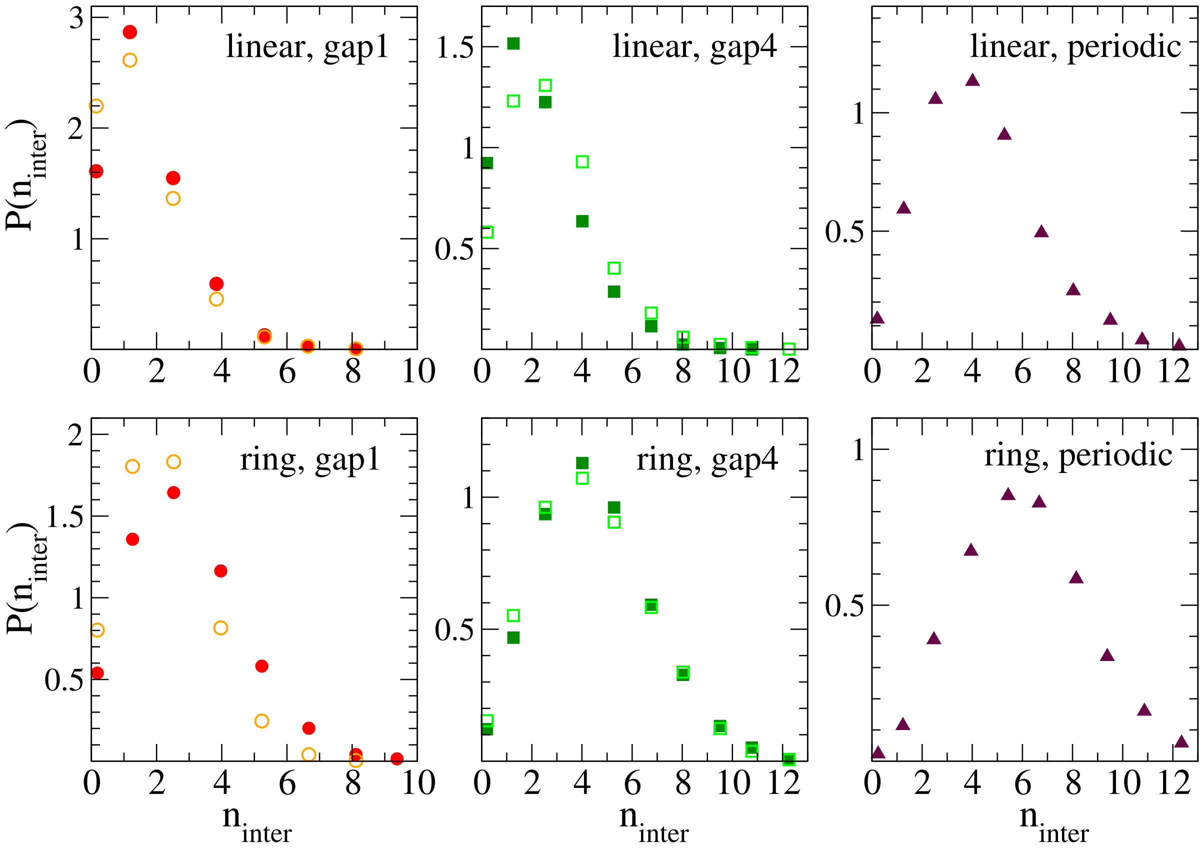

Figure S9. Distribution of instantaneous values of the number of intermolecular bonds for two interacting polymers (linear-linear and ring-ring) with reversible bonds and different sequences of reactive sites, at a mutual distance $r = 3\sigma$ corresponding to the maximum of the effective force. For the 'gap1' and 'gap4' cases, the two data sets corresponds to the respective sequences 'couple1' (filled symbols) and 'couple2' (empty symbols).

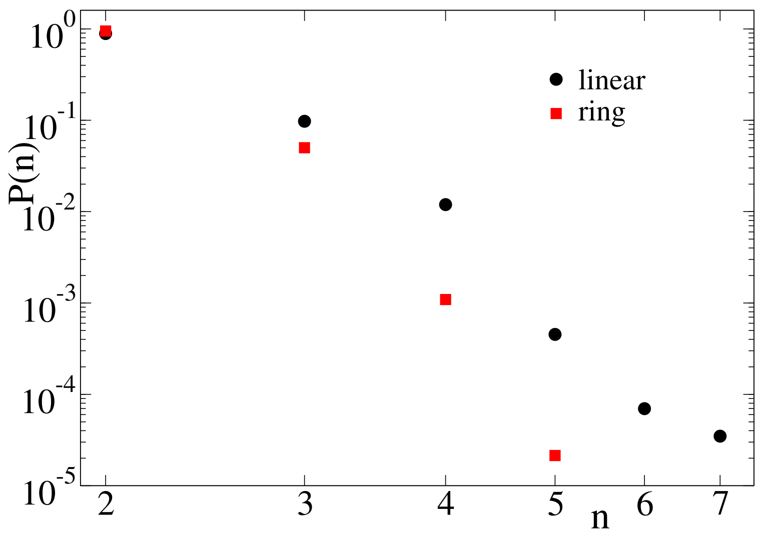

Figure S10. Cluster size distribution in the pure solutions of linear chains and rings with reversible bonds, at the lowest investigated concentration. The cluster size is the number of polymers in the cluster. Two polymers belong to the same cluster if they are mutually linked by at least one intermolecular bond.

References

(1) Henderson, D. *Fundamentals of Inhomogeneous Fluids*; Marcel Dekker, Inc., New York, 1992.

(2) Chubak, I.; Locatelli, E.; Likos, C. N. *Mol. Phys.* **2018**, *116*, 2911–2926.

(3) Overduin, S. D.; Likos, C. N. *J. Chem. Phys.* **2009**, *131*, 034902.

(4) Louis, A. A.; Bolhuis, P. G.; Hansen, J. P. *Phys. Rev. E* **2000**, *62*, 7961.
